# Profiling of Amino Acids in Urine Samples of Patients Suffering from Inflammatory Bowel Disease by Capillary Electrophoresis-Mass Spectrometry

**DOI:** 10.3390/molecules24183345

**Published:** 2019-09-14

**Authors:** Juraj Piestansky, Dominika Olesova, Jaroslav Galba, Katarina Marakova, Vojtech Parrak, Peter Secnik, Peter Secnik, Branislav Kovacech, Andrej Kovac, Zuzana Zelinkova, Peter Mikus

**Affiliations:** 1 Department of Pharmaceutical Analysis and Nuclear Pharmacy, Faculty of Pharmacy, Comenius University in Bratislava, Odbojarov 10, SK-832 32 Bratislava, Slovak Republic; 2 Toxicological and Antidoping Center, Faculty of Pharmacy, Comenius University in Bratislava, Odbojárov 10, SK-832 32 Bratislava, Slovak Republic; 3 Institute of Neuroimmunology, Slovak Academy of Science, Dubravska cesta 9, SK-845 10, Bratislava, Slovak Republic; 4 SK-Lab s.r.o., Partizanska 15, SK-984 01, Lucenec, Slovak Republic; 5 Department of Gastroenterology, St Michael’s Hospital, Satinskeho 1, SK-811 08 Bratislava, Slovak Republic

**Keywords:** inflammatory bowel disease, amino acids profiling, clinical analysis, human urine, capillary zone electrophoresis, mass spectrometry

## Abstract

Urine represents a convenient biofluid for metabolomic studies due to its noninvasive collection and richness in metabolites. Here, amino acids are valuable biomarkers for their ability to reflect imbalances of different biochemical pathways. An impact of amino acids on pathology, prognosis and therapy of various diseases, including inflammatory bowel disease (IBD), is therefore the subject of current clinical research. This work is aimed to develop a capillary electrophoresis-tandem mass spectrometry (CE-MS/MS) method for the quantification of the 20 proteinogenic amino acids in human urine samples obtained from patients suffering from IBD and treated with thiopurines. The optimized CE-MS/MS method, with minimum sample preparation (just “dilute and shoot”), exhibited excellent linearity for all the analytes (coefficients of determination were higher than 0.99), with inter-day and intra-day precision yielding relative standard deviations in the range of 0.91–15.12% and with accuracy yielding relative errors in the range of 85.47–112.46%. Total analysis time, an important parameter for the sample throughput demanded in routine practice, was shorter in ca. 17% when compared to established CE-MS methods. Favorable performance of the proposed CE-MS/MS method was also confirmed by the comparison with corresponding ultra-high performance liquid chromatography-mass spectrometry (UHPLC-MS) method. Consistent data for the investigated amino acid metabolome were obtained using both methods. For the first time, the amino acid profiling by CE-MS approach was applied on the clinical IBD samples. Here, significant differences observed in the concentration levels of some amino acids between IBD patients undergoing thiopurine treatment and healthy volunteers could result from the simultaneous action of the disease and the corresponding therapy. These findings indicate that amino acids analysis could be a valuable tool for the study of mechanism of the IBD treatment by thiopurines.

## 1. Introduction

Qualitative and/or quantitative measurement of targeted compounds of metabolism in human body fluids has been established as a crucial tool for the prediction, diagnosis or investigation of the mechanism of various diseases [1,2,3,4]. Amino acids play an important role in cell metabolism and are associated with different functions—some act as neurotransmitters, serve as essential precursors for the synthesis of variety of molecules with enormous importance or regulate key metabolic pathways and processes that are vital to the health, growth, development, reproduction and homeostasis of organisms [5]. Disturbances in amino acids levels may lead to different metabolic diseases or may also indicate disease status. Therefore, the role of amino acids in origin, progression or control of different diseases is now widely studied [6]. As examples the determination of amino acids levels in patients suffering from osteoarthritis [7], urinary tract infection [8], inborn errors of metabolism [9], prostate or bladder cancer [10] or amyotrophic lateral sclerosis [11], can be mentioned.

Inflammatory bowel disease (IBD) is a chronic, idiopathic and relapsing inflammation of the gastrointestinal tract (GIT) with an unknown etiology, which is characterized by diarrhea, electrolyte loss, bleeding and abdominal pain. Generally, amino acids are important for intestinal growth, mucosal integrity and serve as the precursors of metabolically active proteins, glutathione, nitric oxide or polyamines [12], and, therefore, they may have some impact on the IBD status. Very recently, Vidal-Lletjós et al. [13] reported the study on the influence of glutamine and arginine on IBD progression. Liu et al. [12] discussed the role of glutamate, methionine, cysteine, threonine, tryptophan, glycine, histidine in prophylaxis and therapy of IBD. Significantly decreased levels of serine and increased levels of histidine in plasma were reported by Papada et al. [14] for a group of patients with Crohn’s disease and ulcerative colitis, respectively. Another study showed significantly decreased levels of tryptophan in serum of patients with IBD. Here, the levels of tryptophan were much lower in patients with Crohn’s disease than in patients with ulcerative colitis [15]. The above mentioned findings clearly indicate that amino acids analysis could be a valuable tool in the IBD prognosis and therapeutic management that could enhance the efficacy of the therapy.

Amino acids represent a chemically heterogeneous group of molecules which can exist as negatively charged, positively charged or neutral substances. The analysis of such components of a various nature using a single procedure can therefore be problematic and challenging. Capillary electrophoresis (CE) is a separation method characterized by high separation efficiency, short time of analysis, low costs or variability, offers an appropriate possibility for this purpose. Moreover, CE coupled with mass spectrometry (CE-MS) has been demonstrated to be a powerful and attractive analytical tool that enhanced analysis reliability, especially, when monitoring amino acids in different types of matrices. This is due to the high sensitivity and charge-to-mass selectivity offered by CE and structural information provided by MS. These CE-MS attributes allow amino acid analysis without the need for extensive sample pretreatment or the use of a specific amino acid analyzer [16]. Several papers demonstrated valuable CE-MS methods for the bioanalysis or clinical analysis of native (underivatized) amino acids [7,8,9,17,18,19,20,21,22]. The developed methods were characterized as sufficiently robust, accurate and precise, with predicted detection limits (LODs) ranging in the interval of 0.1–14 µM and applicable to human urine, human serum, dried blood spot or cell lysate supernatant. However, to our knowledge, there have not been any publications demonstrating a method capable of profiling all (proteinogenic) amino acids in urine for IBD via CE-MS/MS, to date. Moreover, only few papers deal with the quantitative amino acid analysis in human urine in connection with inflammatory bowel disease and were performed with the use of liquid chromatography-mass spectrometry (LC-MS) [23], nuclear magnetic resonance (NMR) [24,25,26,27,28,29] or gas chromatography-mass spectrometry (GC-MS) approach [14,23].

An aim of our work was to develop capillary zone electrophoresis-electrospray ionization-tandem mass spectrometry (CZE-ESI-MS/MS) method for simple, fast and reliable analysis of 20 proteinogenic amino acids in human urine samples. The CZE-ESI-MS/MS method was optimized, validated and applied to the analysis of clinical samples obtained from patients suffering from IBD. Differences in the levels of amino acids between IBD patients treated with thiopurines and healthy volunteers were evaluated and discussed.

## 2. Results and Discussion

### 2.1. Development of CZE-ESI-MS/MS Method

To develop a CZE-ESI-MS/MS method for the separation and detection of amino acids in human urine matrices, the compatibility of each analytical step (i.e., CZE, ESI and MS/MS) is essential. Therefore, requirements on volatile background electrolyte (BGE) constituents in the CZE step, effective ionization of the analytes in the CE as well as ESI step and proper fragmentation and detection of the analytes in the MS/MS step were considered in the method optimization.

**Figure 1:**
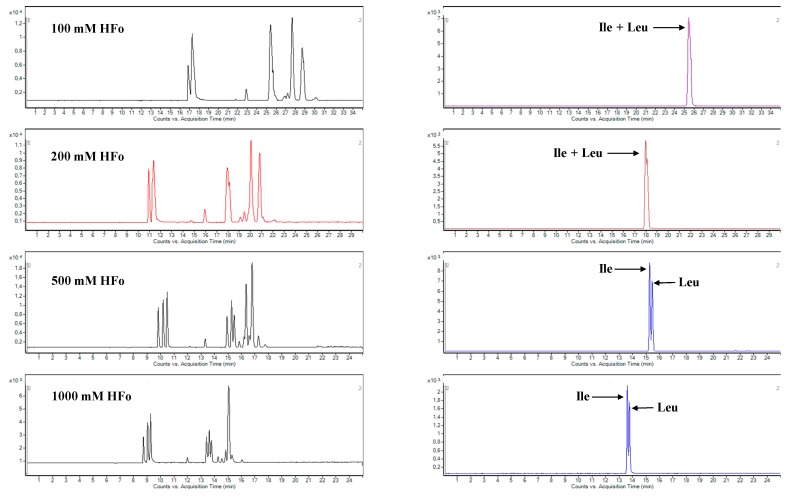
Effect of the concentration of formic acid in background electrolyte (BGE) on the separation selectivity of 20 proteinogenic amino acids in water solution. Left panels: total ion current (TIC) profiles. Right panels: extracted multiple reaction monitoring (MRM) records of two isomeric amino acids (isoleucine and leucine) obtained under the same conditions as TIC. For details of the capillary electrophoresis-tandem mass spectrometry (CE-MS/MS) method see Section 3.

At the beginning of the CZE optimization it was necessary to ensure a proper migration of the all amino acids (of neutral, acidic and basic character) toward to the detector. The application of BGE with a low pH value (below 2.77) can be suitable for this purpose [30]. When applying BGE with the pH value of 1.55, each amino acid was positively charged and migrated toward the detector (electroosmotic flow, EOF, was eliminated under this pH). In order to obtain high intensity and stability of the analytical signal and to resolve all of the amino acids, four concentration levels (100, 200, 500 and 1000 mM) of formic acid (HFo), serving as a BGE constituent, were tested. It was demonstrated that higher concentrations of HFo (and, by that, lower pH values) led to shorter migration times of the analyzed amino acids (see left panels in Figure 1). Moreover, higher concentrations of HFo were essential for the separation of problematic analytes such as isoleucine and leucine isomers that could not be resolved by QqQ MS itself (see right panels in Figure 1).

**Figure 2:**
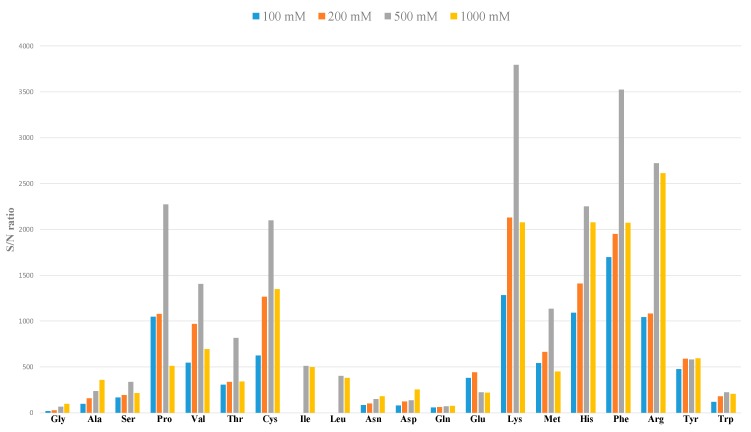
Effect of the concentration of formic acid in BGE on the signal to noise ratio (S/N) of 20 proteinogenic amino acids in water solution. Concentration of all amino acids injected into the electrophoretic analyzer was 100 μM, except from cysteine (50 μM). For the CE-MS/MS method details see Section 3.

On the other hand, when raising the HFo concentration to the value of 1000 mM, a pronounced drop in the intensity of the analytical signal was observed (Figure 2). Therefore, a 500 mM HFo solution was chosen as an optimal separation environment for the proteinogenic amino acids with respect to the best resolution (considering mainly the resolution of isomers, R = 1.10), sensitivity and an excellent stability of the analytical signals (RSD_tm_ 0.24–0.46 %, RSD_area_ 1.55–6.86 %). By comparison, only two papers have documented baseline separation of the isoleucine and leucine isomer pair [7,19]. In both cases, the baseline resolution between the isomers was obtained by addition of a modifier (organic solvent-methanol). The separation of the amino acids in such environment was characterized by the migration time of the slowest analyte at the level approximately 25 min and the total time of such analysis was 30 min. On the other hand, the migration time of the slowest analyte achieved by our CE-MS method was approximately 18 min and the total time of such analysis was 25 min. According to these results it can be stated that our CE-MS method is able to save approximately 17% of the time of analysis and is characterized by a resolution between isoleucine and leucine isomers which is satisfactory for their quantification purposes.

In the electrospray ionization (ESI) step, the composition of sheath liquid was optimized to provide a stable electrical connection between the tip of the capillary and ground and for the effective generation of electrospray and its stability. Sheath liquids with different composition of organic solvents (methanol, isopropanol) and volatile acid (0.1–0.5% HFo) or salt of volatile acid (1–10 mM ammonium acetate, NH_4_Ac) were examined. A deterioration of analytical signal (intensity, peak shape) was observed when using higher amounts of organic solvent. Although higher concentration levels of formic acid in the sheath liquid tended to partially increase the signal intensity of analyzed amino acids, the stability of the analytical signal was decreased. An optimum sheath liquid composition, providing required CZE-ESI compatibility and performance, was methanol/water 50:50 (*v*/*v*) + 5 mM NH_4_Ac.

Other optimized ESI parameters, responsible for the effective ionization of analytes and the generation of stable electrospray, included sheath liquid flow rate (4–10 μL.min^−1^, optimum value 8 μL.min^−1^), nebulizer pressure (5–20 psi, optimum value 10 psi), drying gas temperature (150–350 °C, optimum value 300 °C), drying gas flow rate (3–10 L.min^−1^, optimum value 10 L.min^−1^) and capillary voltage (3000–5500 V, optimum value 4000 V). It was demonstrated that the increase in drying gas temperature, flow rate or capillary voltage led to higher response of the analytical signal but lower stability (reproducibility). The increase in sheath liquid flow rate resulted in decrease in the analytical signal due to the dilution effect; however, lower values were associated with unstable electrospray generation (current drop was observed very often). Similar observations were obtained also for higher or lower levels of nebulizer gas pressure. Hence, optimal values of the ESI parameters were chosen as a compromise between sensitivity and stability, that is, intensity and reproducibility of the analytical signal.

In the MS/MS step, a chronological application of the QqQ software operation modes (Scan mode, Selected ion monitoring (SIM), Product ion mode and multiple reaction monitoring (MRM)) was carried out to find parent ions of the all analytes (see Table 1) and an appropriate value of fragmentor voltage (50–150 V, optimum value 100 V) as well as collision energy and product ions for each of them (see Table 1). The optimized MS/MS parameters provided the parent-product ion transitions being essential for an unequivocal determination and identification of the studied compounds.

### 2.2. Evaluation of Performance Parameters

The optimized CZE-ESI-MS/MS method was validated for particular performance parameters according to the US Food and Drug Administration (FDA) guidance for bioanalytical method validation [31]. The model mixtures of 20 proteinogenic amino acids were prepared in demineralized water and in human urine.

Calibration lines of the all amino acids in water and human urine model matrices, summarized in Table 1 and Table 2, were linear in the tested concentration ranges (exceeding one decadic order) with the determination coefficients ranging in the interval of 0.9958–0.9993 and 0.9897–0.9997, respectively. High separation efficiency (~1 × 10^5^ theoretical plates) and low LOD (0.02–11.03 µM in water and 0.22–8.73 μM in urine) of the method for the tested group of amino acids provided favorable conditions for their separation (prerequisite for Leu and Ile) and quantification.

Selectivity of the method was evaluated by measuring six individual blank and spiked human urine samples (10-times diluted). The required selectivity of the developed method enabled the resolution of the amino acids from each other, and, in addition, from the urine matrix constituents, was achieved thanks to the MS detection (based on monitoring characteristic *m*/*z* values and parent-product ion transitions, for the experimental MS data see Table 1 and Table 2). Here, the CZE separation was beneficial for the time resolution of the analytes, mainly of value for isomer distinction.

The accuracy and precision of the method were evaluated by five replicate analyses of three different quality control (QC) samples (low, medium, high) with known amounts of the analytes. The obtained data are summarized in Table 3. The intra-assay precision (%RSD) for all the analytes ranged from 0.91% to 11.86% and the accuracy (%RE) was in the interval of 85.47–112.46%. Similarly, for the inter-assay precision experiments (for five consecutive validation days) RSD varied from 0.93% to 15.12% and the accuracy from 85.74% to 105.18%. Recovery was evaluated at three different spiked concentration levels of the analytes and in five replications. The percentage recovery was determined by comparing a nominal concentration of the standard with a concentration difference of the same analyte between spiked and non-spiked QC sample (10-times diluted urine). The recovery of the amino acids ranged from 85.07% to 102.05%. Analysis of the QC samples clearly demonstrated acceptable reliability of the proposed CE-MS/MS method as required in practical use.

The stability of the amino acids in the biological human urine matrix was examined by way of the short-term stability and freeze and thaw stability testing. Both tests were performed with the use of three QC samples (low—15 μM (7.5 μM for Cys), medium—150 μM (75 μM for Cys), high—400 μM (200 μM for Cys)). Short-term stability was assessed after 24 h storage of the QC samples at room temperature, freeze and thaw stability testing was performed after three freeze-thaw cycles when the samples were frozen and subsequently thawed at room temperature. The observed relative error did not exceed 11.50%. Results from these experiments are summarized in Table 4 and they clearly indicate acceptable stability of the tested analytes (no significant difference was observed between nominal and found concentrations of monitored amino acids).

The comparative ultra-high performance liquid chromatography-mass spectrometry (UHPLC-MS) method was characterized by excellent linearity within the tested concentration range (1–500 μM and 0.5–250 μM for cysteine) and favorable LOD and LLOQ values ranging in the interval of 0.072–3.87 μM and 0.5–5 μM, respectively. These parameters were satisfactory for the quantitation of amino acids in the studied clinical human urine samples. Moreover, the UHPLC-MS represents a very fast analytical tool for the analysis of amino acids—the total time of analysis was 10.5 min (plus 10 min of the derivatization procedure) and the retention time of the last eluting amino acid (tryptophan) was only 5.59 min.

According to the aforementioned findings obtained from measurements of model biological samples, it can be stated that the developed and validated CE-MS/MS method is suitable for the identification and quantification of 20 amino acids in human urine and can be transferred into the field of real clinical samples. It represents an economic alternative to the UHPLC-MS approach with comparable total analysis time. The CZE technique has an evident advantage over the UHPLC techniques as to the volume of the dosed sample (nL vs. μL) and small amount of the produced organic waste (methanol/water/NH_4_Ac sheath liquid with the flow rate of 8 μL.min^−1^ vs. acetonitrile/water/formic acid mobile phase with the flow rate 500 μL.min^−1^). Moreover, economic benefits can be seen not only in the basic equipment (electrophoretic analyzer vs. UHPLC analyzer) but also in the separation compartments used. For example, a bare fused silica capillary, used in the CE technique as the separation column, is much cheaper than UHPLC columns. Thus, CE-MS represents a favorable alternative to UHPLC-MS for clinical routine amino acids profiling.

### 2.3. Method Application

The developed and validated CE-MS/MS method was applied to the determination of amino acids in clinical human urine samples. They were obtained from thirteen volunteers (4 men and 9 women) suffering from Crohn’s disease which gave their informed consent before they participated in the study. All the patients were treated with the azathioprine. The dosage was 50–125 mg per day. The group of ten healthy volunteers (5 females, 5 males) serves as a control, representing the endogenous levels of free amino acids in urine (being in the linear concentration range as given in Table 1). The amino acids endogenous levels measured in our work (see Supplementary Material, Table S1) are in a good agreement (including also low concentration levels of Pro) with those published in the literature [32,33]. All the samples used in our work were collected at the Department of Gastroenterology of Saint Michael’s Hospital in Bratislava.

**Figure 3:**
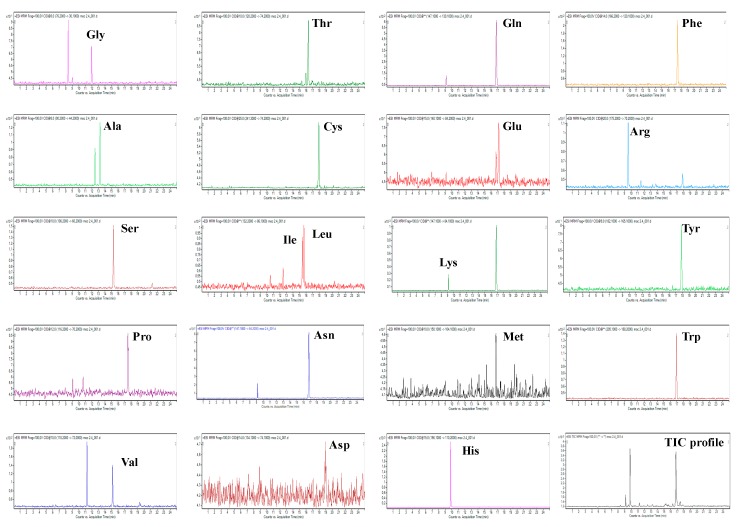
Extracted analytical profiles of clinical sample. Multiple reaction monitoring (MRM) transitions from the CE-MS/MS analysis of a 10-fold diluted human urine sample obtained from a patient suffering from inflammatory bowel disease undergoing treatment with thiopurines (azathioprine). For the sample preparation and other working conditions, see Section 3.

Illustrative extracted ion electropherograms of amino acids present in a clinical human urine sample obtained by the CE-MS/MS method are shown in Figure 3.

The measured amino acid concentrations were corrected (normalized) to the concentration of creatinine and resulting data are summarized in Supplementary Material, Table S2. The urinary creatinine concentration is used as a standardization tool for the quantitative evaluation of urinary concentrations of other chemical substances—drugs, xenobiotics or endogenous substances in urine due to the urine dilution effects. Urinary excretion of creatinine is constant between different individuals, within an individual over time. Hence, any fluctuations in the excreted urine (and, by that, amino acids) concentrations can be effectively corrected with the use of corresponding creatinine levels [34]. The CE-MS/MS results were compared with those obtained using the UHPLC-MS method. Selected ion chromatograms of derivatized amino acids provided by the UHPLC-MS analysis of clinical human urine samples obtained from an IBD patient treated with thiopurines and from a healthy control are presented in Supplementary Material, Figure S1. The resulting concentrations of the amino acids (normalized on the creatinine concentration) are summarized in the Supplementary Material, Table S3. The clinical data obtained from both methods were consistent and very good correlation between these two datasets was demonstrated via correlation coefficients ranging in the interval of r = 0.9929–0.9997. This comparison clearly confirmed high reliability of the clinical data produced by the developed CE-MS/MS method and its usefulness for routine use.

**Figure 4:**
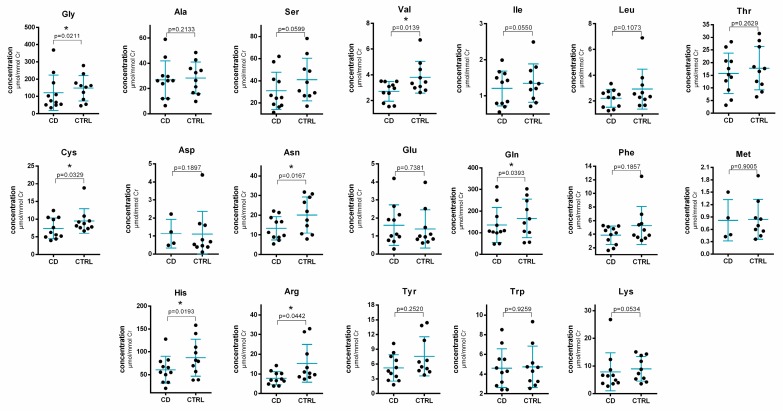
Statistical evaluation of the changes in amino acid concentrations in human urine samples obtained from inflammatory bowel disease (IBD) patients (CD) undergoing thiopurine treatment and from healthy control subjects (CTRL). For the sample preparation and other working conditions, see Section 3. The asterisk (*) indicates a statistical significance on the level *p* < 0.05.

Statistical evaluation of the changes in amino acid concentrations in human urine samples obtained from IBD patients treated with thiopurines and from healthy control subjects is illustrated in Figure 4.

A moderate decrease of seven amino acids, namely Gly, Val, Cys, Gln, Asn, His, Arg, in IBD patients was observed. The concentration decrease was interpreted with significance *p* < 0.05. Some of the previous studies based on the NMR or GC-MS analytical platforms identified no difference between IBD (treated with thiopurines and/or with other drugs) and healthy control in amino acids urinary levels [26,28]. On the other hand, other studies demonstrated elevated concentrations of Gly [24,27], decreased concentrations of Ala [35], Asn, Lys and His [25] in active IBD patients (treated with thiopurines and/or with other drugs) and down-regulated Gly and Ala in the case of IBD patients in remission [27]. Only one of the abovementioned studies deals also with the evaluation of IBD patients (relatively small group of 25 individuals) who were taking no medication. The same effect as in the case of patients undergoing thiopurine treatment (i.e., increased concentrations of Gly and unchanged levels of other amino acids) was observed also in the comparison to healthy controls [24]. Martin et al. [23], dealing with the analysis of urine samples from pediatric IBD patients (treated with thiopurines and/or other drugs), reported significantly higher levels of Glu, Gly and Cys. The decreased levels of Asn, Lys and His could potentially be related to the intestinal malabsorption caused by the disease [25].

## 3. Materials and Methods

### 3.1. CE-MS/MS Instrumentation and Separation Conditions

All experiments were carried out on an Agilent 7100 capillary electrophoresis system (Agilent Technologies, Santa Clara, CA, USA) coupled with an Agilent 6410 Series Triple Quadrupole tandem mass spectrometer (Agilent Technologies, Santa Clara, CA, USA) through an electrospray (ESI) interface. The CE data acquisition and system control were performed with Agilent ChemStation B.04.03 software.

Capillary electrophoresis separations were carried out in a 50 µm inner diameter (I.D.) × 300 µm outer diameter (O.D.) × 900 mm length fused silica capillary (MicroSolv Technology Corporation, Eatontown, NJ, USA). Before the first use, a new capillary was rinsed and conditioned for 10 min with 1 M sodium hydroxide, for 10 min with deionized water and for 10 min with the background electrolyte (BGE). At the end of each day, the capillary was rinsed with 0.1 M sodium hydroxide for 10 min, deionized water for 20 min and BGE for 10 min and stored in BGE overnight. The sample was inserted hydrodynamically with a pressure of 50 mbar for 10 s. The separation was performed in a cationic separation regime, the applied voltage was set at + 30 kV. Before each injection the capillary was re-equilibrated by applying of negative voltage −20 kV for 30 s and flushing with BGE for 2 min.

An on-line coupling of the CE instrument with the Agilent 6410 Series Triple Quadrupole tandem mass spectrometer was carried out through a coaxial sheath-flow ESI interface. The system control and data acquisition were performed with Mass Hunter Work Station B.03.01 software (Agilent Technologies, Santa Clara, CA, USA). The Agilent 1260 Infinity LC pump (Agilent Technologies, Santa Clara, CA, USA) equipped with a 1:100 splitter was used to deliver the sheath liquid in to the CE interface. ESI-MS was conducted in the positive ion mode and the capillary voltage was set at 4000 V. Capillary temperature was set at 300 °C. Nitrogen was used as a drying and nebulizing gas and was delivered to the outer capillary channel under the pressure of 10 psi and the flow of 10 L/min. The equipment was operated at the multiple reaction monitoring (MRM) mode using characteristic precursor ion-product ion mass transitions for each amino acid. Dwell time was 50 ms.

### 3.2. UHPLC-MS Instrumentation and Separation Conditions

For the comparison, alternative UHPLC-MS method was applied to monitor amino acids profiles. All of the analyses were carried out on the chromatographic apparatus Acquity UPLC H-Class equipped with a quaternary gradient pump, autosampler and column thermostat (Waters, Prague, Czech Republic). The UPLC apparatus was coupled with the single quadrupole mass spectrometer—QDa (Waters, Prague, Czech Republic), equipped with the electrospray (ESI) ionization source operated in a positive ionization mode. The following MS parameters were used: capillary voltage 0.8 kV, source block temperature 150 °C, probe temperature 600 °C, cone voltage 15–30 V. A computer with the MassLynx software (Waters, Prague, Czech Republic) was used for the acquisition and processing of data from the LC-MS apparatus. The analytes and their deuterated internal standards were monitored according to their specific *m*/*z* value in the single ion-monitoring (SIM) mode. Detail information about the mass spectrometry condition for derivatized amino acids is described in Table S4.

The chromatographic separation of twenty amino acids was carried out according to our previously developed LC-MS method [36]. Briefly, the separation was carried out using a reversed phase column Cortecs UPLC C18 (2.1 mm × 100 mm, 1.6 µm) obtained from Waters (Prague, Czech Republic). The mobile phase A consisted of 0.1% formic acid in LC-MS grade water and the mobile phase B consisted of 0.1% formic acid in acetonitrile. The following gradient elution was used: 1% B (0–0.7 min), increased to 13% B (0.7–1.3 min), to 15% B (1.3–3.7 min), to 40% B (3.7–7.0 min), to 95% B (7.0–8.0 min), hold on 95% B (8.0–9.0 min), returning to 1% B (9.0–9.8 min) and re-equilibrating (9.8–10.5 min). The flow rate of the mobile phase was set at 0.5 mL.min^−1^. During all of the analyses the column temperature was maintained at 55 °C. The injection volume was 1 µL.

### 3.3. Chemicals and Samples

A commercial amino acids standard mixture, obtained from Sigma Aldrich (Steinheim, Germany), included 17 L-amino acids at a concentration of 2.5 mM, except from cysteine at 1.25 mM, in 0.1 M HCl. L-asparagine, L-glutamine and L-tryptophan were purchased separately (Sigma Aldrich, Steinheim, Germany) since they were not included in the commercial standard mixture. Isotopically labeled standards of L-amino acids for use as internal standards were obtained from Cambridge Isotope Laboratories (Tewksbury, MA, USA).

LC-MS grade chemicals used for the preparation of the electrolyte solutions and sheath liquid were purchased from Merck (Darmstadt, Germany), Sigma Aldrich (Steinheim, Germany) and Fluka (Buchs, Switzerland). Demineralized water prepared with the aid of a water purification system Millipore Simplicity 185 (UV) (Millipore, Molsheim, France) was used as a solvent for the electrolytes, sheath liquid and samples. The electrolytes were filtered before use through disposable membrane filters (0.22 μm pore size, Millipore, Molsheim, France) and were stored in the fridge before analysis.

The derivatization reagent AccQTag Ultra and the derivatization buffer AccQTag Ultra borate buffer (pH = 8.6) for UHPLC-MS experiments were from Waters Corporation (Prague, Czech Republic).

### 3.4. Procedures for Sample and Standard Solution Preparation

#### 3.4.1. Standard Solutions, Calibration Solutions and Quality Control (QC) Samples

Individual stock solutions of three amino acids (Asn, Gln, Trp), which were not included in the commercial amino acids standard mixture, were prepared by dissolving of an appropriate amount of each solid substance in 0.1 M HCl at following concentration levels: glutamine—3.8 mg/mL (26 mM), asparagine—3 mg/mL (22.71 mM) and tryptophan—3 mg/mL (14.69 mM).

The final stock solution mixture of all 20 amino acids was prepared by mixing of the individual stock solutions of amino acids and their appropriate dilution with 0.1 M HCl obtaining 500 µM concentration levels (except from 250 µM cysteine) of the individual amino acids in the mixture. Working solutions (calibration, QC) were made by a proper dilution of the final stock solution mixture with demineralized water or by mixing a proper amount of the final stock solution mixture with properly diluted human urine.

Concentration levels of amino acids in the injected calibration solutions were in the range of 3 to 500 µM (6.25–250 µM for cysteine). Each calibration point was measured 5 times. Parameters of calibration lines of the analytes were calculated by using Microsoft Excel 2007 (Microsoft Corporation, Redmond, WA, USA).

Quality control samples (QCs) were prepared in 10-fold diluted human urine at three concentration levels—15 (low), 150 (medium) and 400 (high) µM for each of 19 amino acids except from 7.5 (low), 75 (medium) and 200 (high) µM for cysteine. Each QCs concentration point was measured 5 times.

#### 3.4.2. Urine Sample Collection and Preparation

Thirteen clinical samples of human urine were obtained from patients suffering from Crohn’s disease who were being treated with azathioprine (9 females, 4 males—detail data described in Supplementary Material Table S5) and ten samples of human urine were obtained from healthy volunteers (5 females, 5 males, age in the interval of 24–46). All the subjects participating in this study gave their informed consent for inclusion before the collection of the samples. Each urine sample was frozen (−18 °C) immediately after the sampling and kept in the freezer until use.

Before CE-MS/MS analysis, the samples were thawed out just before the analysis, 10-fold diluted with demineralized water, filtered through disposable membrane filters (0.22 μm pore size, Millipore, Molsheim, France) and immediately injected to the electrophoretic analyzer.

Before UHPLC-MS analysis, a 10 µL volume of each human urine sample was transferred into the 1.5 mL Eppendorf tube to which 10 µL of LC-MS grade water and 5 µL of the internal standard mixture were added. Proteins were precipitated using 40 µL methanol with 0.1% formic acid (*v*/*v*) and centrifuged (13,000× *g*, 10 min). Thereafter, a 10 µL volume of the supernatant was transferred to a vial for derivatization. The derivatization procedure was performed with the use of the AccQTag Ultra derivatization agent. The derivatization agent was prepared in the following way: 1 mL of acetonitrile was added to the vial with AccQTag Ultra reagent powder, vortexed and dissolved by heating at 55 °C for 15 min. Then, 70 µL of borate buffer (pH = 8.6) was added to the samples followed by 20 µL of the prepared AccQTag Ultra derivatizing reagent solution, vortex mixing and heating at 55 °C for 10 min. Samples were then transferred in to the vials and analyzed.

### 3.5. Creatinine

Creatinine analysis was performed by the clinical laboratory SK-Lab (Lucenec, Slovakia) using an enzymatic procedure with the use of the Dimension Vista^®^ 1500 system (Siemens Healthcare, Erlangen, Germany). In a coupled enzyme reaction, creatininase hydrolyzes creatinine to creatine, which is hydrolyzed by creatinase to sarcosine. Sarcosine oxidase hydrolyzes sarcosine to glycine, formaldehyde and peroxide. The peroxide and a chromogen in the presence of peroxidase form a colored end product that is proportional to the amount of creatinine in the sample. The colored reaction product is measured at 540 and 700 nm. The analytical measurement range of the creatinine in urine was 250–35,400 μM. The human urine samples were measured directly from the specimen without any pretreatment–except dilution (see Table S6).

### 3.6. Statistical Data Analysis

The statistical analyses were performed using GraphPad Prism 8.0.2 (GraphPad Software, San Diego, CA, USA). Differences between means were analyzed using two-tailed Student’s *t*-test. For correlation, Pearson´s correlation coefficient was used. Differences at *p* < 0.05 were accepted as statistically significant. * *p* < 0.05, ** *p* < 0.01, *** *p* < 0.001 are used to denote statistical significance.

## 4. Conclusions

In conclusion, we developed and validated a simple, fast, economical and effective method for the quantification of 20 proteinogenic amino acids in human urine—a matrix advantageous for its noninvasive collection. Benefits of the proposed method (compared to the previously reported CE-MS or LC-MS) include enhanced sample throughput due to shorter analysis time, no sample pretreatment (only simple dilution of the sample—“dilute and shoot”), no need for derivatization procedure and acceptable resolution of isobaric amino acids (Leu, Ile) necessary for quantification purposes. Favorable performance and reliability of the proposed CE-MS/MS method, additionally confirmed by the comparative UHPLC-MS analysis, are attributes demanded in routine clinical analysis. In this work, the both developed methods reflected in the successful profiling of the clinical samples obtained from 13 IBD patients.

Statistical evaluation revealed some significant changes in amino acid concentrations in the samples obtained from IBD patients undergoing thiopurine treatment compared to healthy control subjects. The results of this study are encouraging as our findings (i.e., concentration differences) are in agreement with several of the previously published works and additional amino acids (Val, Gln, Arg) were detected as new potential markers, possibly useful for understanding the IBD treatment mechanism. The statistical significance of the results is limited by the number of IBD patients included in this pilot study. In addition, for a more detailed study of the IBD treatment mechanism, a group of IBD patients that are not being influenced by any therapy should also be included. Therefore, in our future work, higher numbers of IBD patients, as well as patients before and during thiopurine treatment, will be considered. Moreover, for other clinical studies (IBD diagnostics, drugs interactions and synergy, etc.), the exact stratification of the patients in groups, for example, with active IBD, IBD in remission, responding or non-responding patients, mono or combined therapy, will be considered as well. Such an approach may provide profound insights into the metabolic changes associated with IBD, with utilization in IBD diagnostics or therapy optimization.

## Figures and Tables

**Table 1:** Calibration and selected analytical parameters of the CE-MS/MS method for 20 amino acids in model water matrix.

Analyte	Parent Ion–Product Ion Transition (*m*/*z*)	Range (µM)	Calibration Line	Linearity (r^2^)	LOD (µM)	LLOQ (µM)	t_m_ (min)	N
**Glycine**	76.2→30.1	15–500	y = 3.3694x + 1.5964	0.9965	11.03	18.38	10.63	111,289
**Alanine**	90.2→44.2	15–500	y = 35.668x − 37.131	0.9992	1.33	2.22	11.53	101,866
**Serine**	106.2→60.2	3–500	y = 33.646x + 142.79	0.9987	2.44	4.06	12.90	121,801
**Proline**	116.2→70.2	3–500	y = 424.642x + 108.53	0.9974	0.63	1.05	14.31	260,363
**Valine**	118.2→72.0	3–500	y = 168.106x − 133.02	0.9965	0.70	1.16	12.81	109,815
**Threonine**	120.2→74.2	15–500	y = 34.036x + 1.223	0.9958	1.71	2.85	13.57	117,865
**Cysteine**	241.3→74.2	6.25–250	y = 50.700x − 49.235	0.9986	0.47	0.79	14.56	98,824
**Isoleucine**	132.2→69.2	3–500	y = 30.944x − 34.896	0.9992	1.33	2.22	13.00	123,659
**Leucine**	132.2→86.1	3–500	y = 198.105x − 28.692	0.9963	1.57	2.61	13.11	106,688
**Asparagine**	133.1→74.0	3–500	y = 12.194x + 23.981	0.9973	5.19	8.65	14.86	79,716
**Aspartic acid**	134.1→74.1	15–500	y = 27.142x − 36.341	0.9930	7.14	11.91	15.19	190,107
**Glutamine**	147.1→84.1	15–500	y = 15.483x + 94.291	0.9935	7.48	12.47	13.78	81,339
**Lysine**	147.1→84.2	3–500	y = 216.476x + 34.134	0.9973	0.38	0.64	8.54	82,399
**Glutamic acid**	148.1→84.2	3–500	y = 101.390x − 34.946	0.9983	1.91	3.19	14.07	126,768
**Methionine**	150.1→104.1	3–500	y = 47.415x + 8.5213	0.9975	0.50	0.84	13.82	124,994
**Histidine**	156.1→110.2	15–500	y = 306.972x + 34.669	0.9988	0.02	0.04	9.02	91,906
**Phenylalanine**	166.2→120.1	3–500	y = 539.879x − 206.72	0.9993	0.38	0.64	14.24	119,378
**Arginine**	175.2→70.2	3–500	y = 236.186x + 78.621	0.9969	0.34	0.57	8.82	85,396
**Tyrosine**	182.1→165.1	3–500	y = 70.794x + 15.698	0.9992	1.08	1.8	14.47	113,759
**Tryptophan**	205.1→118.1	3–500	y = 68.235x + 107.91	0.9984	1.92	3.21	14.01	91,491

**Table 2:** Calibration and selected analytical parameters of the CE-MS/MS method for 20 amino acids in human urine matrix.

Analyte	Parent Ion–Product Ion Transition (*m*/*z*)	Range (µM)	Calibration Line	Linearity (r^2^)	LOD (µM)	LLOQ (µM)	t_m_ (min)	N
**Glycine**	76.2→30.1	15–500	y = 2.6075x + 25.297	0.9976	5.45	9.08	11.96	95,241
**Alanine**	90.2→44.2	15–500	y = 30.587x + 429.8	0.9997	1.62	2.71	13.24	97,838
**Serine**	106.2→60.2	3–500	y = 26.391x + 247.5	0.9983	2.61	4.36	15.30	117,903
**Proline**	116.2→70.2	3–500	y = 269.53x + 493.84	0.9984	0.40	0.66	17.51	99,395
**Valine**	118.2→72.0	3–500	y = 126.42x + 165.05	0.9994	0.51	0.85	15.18	113,496
**Threonine**	120.2→74.2	15–500	y = 29.596x + 266.15	0.9995	1.31	2.18	16.35	92,125
**Cysteine**	241.3→74.2	6.25–250	y = 43.708x + 25.297	0.9993	0.31	0.52	17.96	99,185
**Isoleucine**	132.2→69.2	3–500	y = 135.27x + 20.916	0.9991	0.23	0.38	15.45	156,381
**Leucine**	132.2→86.1	3–500	y = 114.7x + 92.705	0.9979	0.93	1.54	15.68	116,776
**Asparagine**	133.1 →74.0	3–500	y = 14.272x + 241.43	0.9968	1.24	2.07	16.33	80,394
**Aspartic acid**	134.1→74.1	15–500	y = 23.42x + 291.32	0.9995	8.73	14.56	16.69	102,863
**Glutamine**	147.1→84.1	15–500	y = 19.263x + 908.9	0.9897	1.37	2.28	16.81	93,292
**Lysine**	147.1→84.2	3–500	y = 213.13x + 161.59	0.9944	0.45	0.75	9.14	97,211
**Glutamic acid**	148.1→84.2	3–500	y = 109.51x + 210.19	0.9984	1.45	2.42	17.16	83,677
**Methionine**	150.1→104.1	3–500	y = 40.467x + 29.661	0.9993	0.63	1.04	16.77	106,854
**Histidine**	156.1→110.2	15–500	y = 293.33x + 700.5	0.9994	0.33	0.55	9.82	84,402
**Phenylalanine**	166.2→120.1	3–500	y = 376.93x + 342.59	0.9991	0.32	0.53	17.45	101,095
**Arginine**	175.2→70.2	3–500	y = 189.47x + 97.003	0.9992	0.22	0.37	9.56	86,366
**Tyrosine**	182.1→165.1	3–500	y = 41.35x + 76.958	0.9980	1.39	2.32	17.86	95,190
**Tryptophan**	205.1→118.1	3–500	y = 81.434x + 425.82	0.9993	0.84	1.40	16.96	100,132

**Table 3:** Accuracy, precision and recovery data from quality control (QC) samples.

Analyte (QC Level)	Intra-Assay (n = 5)			Inter-Assay (n = 15)			Recovery (%)
	Mean Found Concentration (µM)	RSD (%)	RE (% Nom.)	Mean Found Concentration (µM)	RSD (%)	RE (% Nom.)	
**Glycine**							
QC low	16.01	5.92	6.74	14.97	5.80	−0.19	87.40
QC medium	147.29	5.47	−1.81	140.76	6.65	−6.16	92.31
QC high	380.42	6.01	−4.90	386.94	1.62	−3.26	101.99
**Alanine**							
QC low	14.28	9.70	−4.81	15.10	9.03	0.62	90.87
QC medium	147.36	3.57	−1.76	145.97	3.50	−2.69	90.49
QC high	407.07	2.32	1.77	411.73	3.99	2.93	94.50
**Serine**							
QC low	14.20	2.60	−5.32	14.62	4.61	−2.53	94.55
QC medium	153.23	1.17	2.15	149.90	4.55	−0.06	98.32
QC high	403.69	2.41	0.92	404.24	6.73	1.06	95.54
**Proline**							
QC low	13.56	2.25	−9.63	13.78	2.77	−8.17	93.44
QC medium	151.94	3.54	1.29	151.50	6.22	1.00	92.82
QC high	399.40	4.92	−0.15	402.24	5.88	0.56	94.03
**Valine**							
QC low	14.32	1.26	−4.53	14.53	2.30	−3.15	95.54
QC medium	153.54	2.53	2.36	144.69	8.61	−3.54	95.06
QC high	406.49	3.12	1.62	391.09	5.34	−2.23	95.66
**Threonine**							
QC low	15.57	2.06	3.76	15.48	2.55	3.17	89.61
QC medium	151.58	2.00	1.06	149.93	8.50	−0.05	96.39
QC high	405.95	2.57	1.49	394.41	3.97	−1.40	96.75
**Cysteine**							
QC low	7.95	4.19	5.97	7.19	10.55	−4.12	95.37
QC medium	79.33	2.83	5.78	77.27	5.86	3.02	101.25
QC high	206.96	0.91	3.48	204.89	0.93	2.44	99.99
**Isoleucine**							
QC low	14.58	4.49	−2.80	14.18	6.85	−5.50	96.91
QC medium	148.98	3.87	−0.68	144.84	3.35	−3.44	92.57
QC high	402.74	4.38	0.69	392.06	5.31	−1.99	93.42
**Leucine**							
QC low	14.32	3.67	−4.55	15.17	6.92	1.10	93.93
QC medium	153.09	3.54	2.06	140.17	11.32	−6.55	94.89
QC high	428.14	2.88	7.03	409.89	6.84	2.47	97.88
**Asparagine**							
QC low	13.87	3.62	−7.53	14.37	11.38	−4.23	97.34
QC medium	149.27	9.66	−0.48	142.63	15.12	−4.92	95.17
QC high	384.25	9.87	−3.94	368.74	11.58	−7.81	95.08
**Aspartic acid**							
QC low	14.77	11.86	−1.57	15.27	14.19	1.80	96.61
QC medium	135.74	9.66	−9.51	143.26	12.89	−4.49	92.55
QC high	388.46	4.31	−2.89	394.17	8.20	−1.46	97.08
**Glutamine**							
QC low	15.74	5.73	4.91	15.78	6.27	5.18	91.39
QC medium	168.69	0.55	12.46	146.84	8.48	−2.11	98.13
QC high	403.84	4.89	0.96	385.69	4.92	−3.58	101.07
**Lysine**							
QC low	13.15	3.18	−12.37	14.36	13.13	−4.27	85.07
QC medium	148.80	0.54	−0.80	140.64	6.44	−6.24	92.77
QC high	415.27	0.91	3.82	391.76	7.13	−2.06	93.50
**Glutamic acid**							
QC low	13.10	8.82	−12.67	14.14	12.52	−5.76	85.37
QC medium	136.29	4.59	−9.14	132.94	4.45	−11.37	94.66
QC high	396.10	5.53	−0.98	383.54	10.43	−4.12	97.82
**Methionine**							
QC low	14.53	6.01	−3.14	13.75	10.14	−8.33	102.05
QC medium	163.29	3.63	8.86	148.17	8.65	−1.22	100.27
QC high	407.77	3.10	1.94	381.27	8.36	−4.68	98.70
**Histidine**							
QC low	15.32	1.39	2.14	15.37	1.09	2.43	97.01
QC medium	145.08	2.97	−3.28	143.03	2.87	−4.65	97.51
QC high	395.78	4.89	−1.06	386.85	2.15	−3.29	96.18
**Phenylalanine**							
QC low	14.19	1.71	−5.42	14.74	6.88	−1.73	96.97
QC medium	149.71	3.93	−0.19	141.60	5.00	−5.60	94.12
QC high	402.79	2.11	0.70	395.23	2.71	−1.20	96.29
**Arginine**							
QC low	12.82	2.45	−14.53	14.22	14.06	−5.22	86.30
QC medium	141.78	2.36	−5.48	139.90	2.54	−6.74	93.76
QC high	399.97	3.83	−0.01	392.12	4.21	−1.97	96.52
**Tyrosine**							
QC low	14.46	2.20	−3.63	12.86	10.63	−14.26	89.19
QC medium	160.07	1.93	6.71	148.59	10.78	−0.94	97.28
QC high	421.06	3.42	5.26	403.01	6.44	0.75	97.25
**Tryptophan**							
QC low	14.92	2.93	−0.54	14.22	6.84	−5.22	97.04
QC medium	154.52	3.49	3.01	146.82	7.57	−2.12	100.37
QC high	381.42	3.45	−4.64	394.32	5.66	−1.42	98.35

RE (% Nom)—Relative error (percentage difference from the nominal value). QC low—15 μM (7.5 μM for Cys), QC medium—150 μM (75 μM for Cys), QC high—400 μM (200 μM for Cys).

**Table 4:** Stability testing of 20 amino acids in QC samples.

	Room Temperature Stability (24 h)						Freeze-Thaw Stability (3 cycles)					
	QC Low		QC Medium		QC High		QC Low		QC Medium		QC High	
Analyte	Found (μM)	Accuracy (%RE)	Found (μM)	Accuracy (%RE)	Found (μM)	Accuracy (%RE)	Found (μM)	Accuracy (%RE)	Found (μM)	Accuracy (%RE)	Found (μM)	Accuracy (%RE)
**Gly**	14.64	−2.40	145.32	−3.12	374.30	−6.43	14.79	−1.41	138.42	−7.72	370.53	−7.37
**Ala**	14.12	−5.86	154.73	3.16	386.75	−3.31	14.60	−2.66	145.56	−2.96	357.90	−10.53
**Ser**	14.92	−0.55	157.30	4.87	376.52	−5.87	15.59	3.90	144.91	−3.40	363.74	−9.07
**Pro**	14.86	−0.95	152.39	1.59	388.29	−2.93	15.29	1.92	150.52	0.35	386.73	−3.32
**Val**	15.53	3.53	149.41	−0.39	394.89	−1.28	15.28	1.85	145.22	−3.18	390.97	−2.26
**Thr**	15.02	0.08	155.39	3.59	389.64	−2.59	15.19	1.28	151.65	1.10	388.38	−2.90
**Cys**	7.40	−1.34	77.00	2.67	190.38	−4.81	7.15	−4.69	71.13	−5.17	192.74	−3.63
**Ile**	14.23	−5.16	155.00	3.33	382.15	−4.46	14.30	−4.68	149.28	−0.48	374.61	−6.35
**Leu**	14.10	−6.03	152.15	1.43	393.09	−1.73	15.10	0.64	147.27	−1.82	405.10	1.27
**Asn**	15.44	2.89	161.33	7.56	371.20	−7.20	16.47	9.78	152.48	1.65	366.67	−8.33
**Asp**	13.73	−8.48	148.65	−0.90	385.64	−3.59	14.77	−1.55	160.39	6.93	390.99	−2.25
**Gln**	13.64	−9.07	132.85	−11.44	387.83	−3.04	15.62	4.15	144.06	−3.96	371.99	−7.00
**Glu**	13.39	−10.74	136.96	−8.70	397.61	−0.60	15.69	4.61	133.82	−10.78	376.95	−5.76
**Lys**	13.98	−6.83	142.07	−5.29	361.19	−9.71	14.23	−5.19	142.96	−4.69	388.88	−2.78
**Met**	13.87	−7.54	148.54	−0.97	395.56	−1.11	14.02	−6.55	140.39	−6.41	364.36	−8.91
**His**	15.18	1.16	154.24	2.83	390.41	−2.40	14.80	−1.35	139.13	−7.24	362.44	−9.39
**Phe**	14.96	−0.27	154.91	3.27	386.71	−3.32	15.98	6.49	142.55	−4.97	361.24	−9.69
**Arg**	14.63	−2.50	154.02	2.68	383.41	−4.15	14.81	−1.32	142.20	−5.20	372.78	−6.81
**Tyr**	15.43	2.82	155.02	3.35	413.04	3.26	13.97	−6.90	157.89	5.26	409.93	2.48
**Trp**	14.46	−3.64	149.86	−0.10	393.38	−1.66	13.86	−7.60	138.59	−7.61	377.94	−5.52

## Supplementary Materials

The following are available online: https://www.mdpi.com/1420-3049/24/18/3345/s1, Figure S1: Selected ion chromatograms of derivatized amino acids provided by the UHPLC-MS analysis of clinical human urine samples obtained from a patient suffering from inflammatory bowel disease (left panel) and from a healthy control (right panel). Table S1: Normalized concentrations of amino acids in urine samples from healthy volunteers measured by the CE-MS/MS method. Table S2: Normalized concentrations of amino acids in urine samples from IBD patients undergoing thiopurine treatment measured by the CE-MS/MS method. Table S3: Normalized concentrations of amino acids in urine samples from IBD patients undergoing thiopurine treatment measured by the UHPLC-MS method. Table S4: Mass spectrometric analysis conditions for UHPLC-MS determination of derivatized amino acids. Table S5: Patients’ characteristics. Table S6: Determined creatinine concentrations in the human urine samples.
