# Supplementary material for: Profiling of Amino Acids in Urine Samples of Patients Suffering from Inflammatory Bowel Disease by Capillary Electrophoresis-Mass Spectrometry
Source: Molecules. 2019 Sep 14;24(18):3345. doi: 10.3390/molecules24183345 (PMC6767150; doi:10.3390/molecules24183345)
Supplement: Supplementary file 1 [file molecules-24-03345-s001.pdf]

molecules

Article

Profiling of Amino Acids in Urine Samples of Patients Suffering from Inflammatory Bowel Disease by Capillary Electrophoresis-Mass Spectrometry

Juraj Piestansky [1,2], Dominika Olesova [3], Jaroslav Galba [1], Katarina Marakova [1,2], Vojtech Parrak [3], Peter Secnik [4], Peter Secnik jr. [4], Branislav Kovacech [3], Andrej Kovac [3], Zuzana Zelinkova [5], Peter Mikus [1,2,*]

1 Department of Pharmaceutical Analysis and Nuclear Pharmacy, Faculty of Pharmacy, Comenius University in Bratislava, Odbojarov 10, SK-832 32 Bratislava, Slovak Republic; piestansky@fpharm.uniba.sk (J.P.); galba@fpharm.uniba.sk (J.G.); marakova@fpharm.uniba.sk (K.M.); mikus@fpharm.uniba.sk (P.M.)
2 Toxicological and Antidoping Center, Faculty of Pharmacy, Comenius University in Bratislava, Odbojárov 10, SK-832 32 Bratislava, Slovak Republic
3 Institute of Neuroimmunology, Slovak Academy of Science, Dubravska cesta 9, SK-845 10, Bratislava, Slovak Republic; dominika.olesova@savba.sk (D.O.); branislav.kovacech@savba.sk (B.K.); andrej.kovac@savba.sk (A.K.); vojtech.parrak@gmail.com (V.P.)
4 SK-Lab s.r.o., Partizanska 15, SK-984 01, Lucenec, Slovak Republic; peter.secnik@sklab.sk (P.S.); peter.secnik@infomed.sk (P.S. jr.)
5 Department of Gastroenterology, St Michael's Hospital, Satinskeho 1, SK-811 08 Bratislava, Slovak Republic; zuzana.zelinkova@nsmas.sk (Z.Z.)
* Correspondence: mikus@fpharm.uniba.sk; Tel.: +421-2-50 117 243

(Supplementary Material)

Table of content

Table S1 Normalized concentrations of amino acids in urine samples from healthy volunteers measured by the CE-MS/MS method.

Table S2 Normalized concentrations of amino acids in urine samples from IBD patients undergoing thiopurine treatment measured by the CE-MS/MS method.

Table S3 Normalized concentrations of amino acids in urine samples from IBD patients undergoing thiopurine treatment measured by the UHPLC-MS method.

Table S4 Mass spectrometric analysis conditions for UHPLC-MS determination of amino acids

Table S5 Patients' characteristics

Table S6 Determined creatinine concentrations in the clinical human urine samples

Figure S1 Selected ion chromatograms of derivatized amino acids provided by the UHPLC-MS analysis of clinical human urine samples obtained from a patient suffering from inflammatory bowel disease (left panel) and from a healthy control (right panel).

Table 1. Normalized concentrations of amino acids in urine samples from healthy volunteers measured by the CE-MS/MS method.

	Vol.1	Vol.2	Vol.3	Vol.4	Vol.5	Vol.6	Vol.7	Vol.8	Vol.9	Vol.10
**Glycine**	162.73	178.04	82.32	45.83	54.29	231.49	153.38	279.00	123.21	162.30
**Alanine**	42.75	48.51	16.63	9.79	15.56	34.37	35.88	32.62	26.54	21.38
**Serine**	64.55	78.23	26.44	17.13	26.50	50.56	38.79	48.83	30.89	29.13
**Proline**	<LLOQ	<LLOQ	<LLOQ	<LLOQ	<LLOQ	<LLOQ	<LLOQ	<LLOQ	<LLOQ	<LLOQ
**Valine**	4.42	4.91	2.98	2.08	3.35	3.45	2.63	6.07	3.01	3.78
**Threonine**	28.68	31.54	12.61	6.46	11.52	16.55	17.70	26.24	18.35	8.42
**Cysteine**	7.85	8.14	9.54	7.62	6.07	7.36	10.88	18.08	8.78	9.24
**Isoleucine**	1.07	1.34	0.93	0.71	1.17	1.20	0.82	2.49	1.08	1.38
**Leucine**	3.24	3.81	2.28	1.65	2.34	2.64	1.67	6.09	2.13	2.55
**Asparagine**	30.81	31.75	10.54	7.88	17.57	26.59	14.71	29.01	21.97	10.00
**Aspartic acid**	0.46	0.39	0.39	0.11	0.79	1.69	0.68	1.60	0.53	4.39
**Glutamine**	247.54	302.56	53.70	56.19	164.98	274.05	145.86	207.35	107.15	101.84
**Lysine**	14.33	13.83	5.37	3.56	7.31	15.11	8.84	11.73	5.64	4.24
**Glutamic acid**	1.01	0.95	0.82	0.67	0.99	1.48	0.83	2.52	0.61	3.98
**Methionine**	0.85	1.28	0.56	0.36	0.69	0.88	0.44	1.90	0.60	<LLOQ
**Histidine**	110.00	139.75	56.77	38.63	69.51	79.19	81.49	158.28	97.80	37.96
**Phenylalanine**	5.57	6.94	4.65	3.10	3.55	3.87	5.41	12.52	3.85	3.42
**Arginine**	9.65	13.21	20.15	11.00	7.41	8.38	10.40	33.02	31.39	8.57
**Tyrosine**	10.80	13.85	6.75	3.80	5.10	4.68	6.45	14.42	5.45	4.44
**Tryptophan**	5.13	7.14	4.74	2.85	3.25	2.54	4.69	9.33	4.03	3.32

The amino acid concentrations are expressed as μM/mM of the creatinine.

Table 2. Normalized concentrations of amino acids in urine samples from IBD patients undergoing thiopurine treatment measured by the CE-MS/MS method.

	S1	S2	S3	S4	S5	S6	S7	S8	S9	S10	S11	S12	S13
**Glycine**	50.87	1231.43	N.D.	102.65	49.46	110.19	65.02	179.44	74.49	34.08	54.55	239.36	368.62
**Alanine**	23.94	208.33	N.D.	29.62	6.44	26.35	12.00	26.29	30.18	11.97	26.27	58.94	44.88
**Serine**	18.75	235.26	N.D.	24.23	26.08	39.39	16.33	41.43	24.85	11.51	17.86	62.04	57.26
**Proline**	<LLOQ	<LLOQ	N.D.	<LLOQ	<LLOQ	<LLOQ	1.39	<LLOQ	<LLOQ	<LLOQ	<LLOQ	<LLOQ	<LLOQ
**Valine**	2.61	32.04	N.D.	3.21	1.58	2.57	1.54	3.50	2.95	1.79	3.51	3.52	3.01
**Threonine**	20.67	99.95	N.D.	15.26	14.27	12.84	5.17	17.67	20.05	3.15	9.15	28.21	26.18
**Cysteine**	5.58	42.74	N.D.	4.95	4.41	10.51	3.96	10.54	6.26	5.20	9.91	12.44	7.66
**Isoleucine**	0.79	6.28	N.D.	0.85	0.55	1.38	0.70	1.99	1.67	0.79	1.62	1.43	1.05
**Leucine**	1.53	27.27	N.D.	1.62	2.05	2.25	1.33	2.73	2.28	1.25	2.83	3.36	2.06
**Asparagine**	9.69	177.85	N.D.	8.87	11.27	16.74	5.38	16.61	18.82	8.72	7.24	22.15	21.37
**Aspartic acid**	<LLOQ	<LLOQ	N.D.	<LLOQ	<LLOQ	<LLOQ	0.60	<LLOQ	<LLOQ	<LLOQ	<LLOQ	<LLOQ	<LLOQ
**Glutamine**	99.43	1061.55	N.D.	105.90	108.22	135.08	47.58	175.43	103.69	49.35	110.65	250.09	312.21
**Lysine**	8.66	78.19	N.D.	3.57	6.67	6.70	2.70	12.41	3.92	3.07	4.92	26.85	6.49
**Glutamic acid**	0.96	<LLOQ	N.D.	0.71	0.76	2.65	0.28	2.18	1.01	1.00	4.02	1.91	1.89
**Methionine**	<LLOQ	<LLOQ	N.D.	0.88	<LLOQ	<LLOQ	<LLOQ	<LLOQ	0.47	0.42	<LLOQ	<LLOQ	1.05
**Histidine**	67.41	547.89	N.D.	54.95	55.43	65.44	32.83	82.09	49.95	19.68	33.24	127.50	78.73
**Phenylalanine**	3.17	36.18	N.D.	5.11	2.47	5.23	1.63	4.21	3.94	1.90	4.58	5.03	4.83
**Arginine**	6.34	59.70	N.D.	4.81	3.86	3.82	6.79	11.65	6.59	10.24	10.21	7.45	14.27
**Tyrosine**	1.78	49.85	N.D.	8.32	3.52	7.56	2.62	6.75	5.03	2.69	4.62	4.12	10.18
**Tryptophan**	5.52	34.10	N.D.	7.16	3.17	5.05	2.41	4.16	4.29	2.04	2.81	8.52	4.62

N.D. – not determined (the concentration of creatinine was under the lowest level of the analytical measurement range, i.e. < 250 μM)
The amino acid concentrations are expressed as μM/mM of the creatinine

Table 3. Normalized concentrations of amino acids in urine samples from IBD patients undergoing thiopurine treatment measured by the UHPLC-MS method.

	S1	S2	S3	S4	S5	S6	S7	S8	S9	S10	S11	S12	S13
**Glycine**	50.69	1199.24	N.D.	99.00	47.03	98.71	67.30	154.97	85.83	38.26	53.72	261.18	350.27
**Alanine**	22.07	175.95	N.D.	26.64	7.17	28.92	12.35	29.26	27.96	8.69	16.64	58.03	50.43
**Serine**	23.36	224.81	N.D.	25.86	19.10	33.33	13.98	40.34	22.12	13.18	14.25	69.44	60.27
**Proline**	<LLOQ	<LLOQ	N.D.	<LLOQ	<LLOQ	<LLOQ	1.03	<LLOQ	<LLOQ	<LLOQ	<LLOQ	<LLOQ	<LLOQ
**Valine**	2.08	27.48	N.D.	3.23	1.74	2.71	1.88	4.50	2.87	1.96	3.63	4.07	3.68
**Threonine**	22.64	103.44	N.D.	13.47	14.13	14.46	6.17	15.58	19.37	3.48	7.88	24.28	25.61
**Cysteine**	6.86	62.98	N.D.	4.99	5.49	10.83	3.59	11.68	7.90	5.77	12.12	11.32	8.97
**Isoleucine**	0.79	7.63	N.D.	0.81	0.59	0.83	0.54	1.42	1.35	0.73	1.42	1.51	1.38
**Leucine**	1.88	27.10	N.D.	1.95	1.36	1.71	1.52	3.00	2.25	1.47	2.92	3.43	2.40
**Asparagine**	12.00	122.52	N.D.	10.02	10.35	13.79	5.74	17.53	18.31	8.69	7.43	22.43	22.16
**Aspartic acid**	0.28	14.50	N.D.	0.51	0.59	1.33	0.52	1.24	0.34	0.33	3.10	0.97	1.46
**Glutamine**	103.42	1076.34	N.D.	125.24	108.99	146.08	47.41	169.92	116.31	51.82	114.25	209.47	294.76
**Lysine**	8.60	78.24	N.D.	4.31	5.56	5.83	2.82	14.26	5.81	2.60	4.78	28.98	6.69
**Glutamic acid**	0.92	21.37	N.D.	0.50	0.64	1.63	0.30	2.79	0.99	0.82	3.45	1.48	1.62
**Methionine**	0.73	<LLOQ	N.D.	0.82	0.37	<LLOQ	0.28	<LLOQ	0.71	0.44	<LLOQ	0.93	0.88
**Histidine**	83.98	481.30	N.D.	53.78	39.36	67.46	28.83	84.61	65.25	21.99	27.79	114.42	68.83
**Phenylalanine**	3.61	33.97	N.D.	5.30	2.22	4.38	2.10	5.21	3.85	2.02	3.27	5.63	4.27
**Arginine**	9.49	70.99	N.D.	4.88	3.73	6.00	7.65	11.90	8.21	8.99	12.92	8.01	14.58
**Tyrosine**	1.98	48.47	N.D.	7.29	3.51	7.92	3.03	6.05	4.67	3.12	4.96	4.65	9.43
**Tryptophan**	5.75	23.28	N.D.	6.79	2.94	5.21	2.41	4.68	4.62	2.22	2.39	7.50	4.93

N.D. – not determined (the concentration of creatinine was under the lowest level of the analytical measurement range, i.e. < 250 μM). The amino acid concentrations are expressed as μM/mM of the creatinine.

Table 4. Mass spectrometric analysis conditions for UHPLC-MS determination of derivatized amino acids.

Analyte	MW	*m*/*z* [M +H]$^+$	Cone Voltage (V)
**Glycine***	75.07	246.1	30
**Alanine***	89.09	260.1	30
**Serine***	105.09	276.1	30
**Proline***	115.13	286.1	30
**Valine***	117.15	288.1	30
**Threonine***	119.12	290.1	30
**Cysteine***	121.16	581.0	34
**Isoleucine***	131.18	302.1	30
**Leucine***	131.18	302.1	30
**Asparagine***	132.12	303.1	30
**Aspartic acid***	133.10	304.1	30
**Glutamine***	146.15	317.1	30
**Glutamic acid***	147.13	318.1	30
**Lysine***	146.19	487.1	18
**Methionine***	149.21	320.1	30
**Histidine***	155.16	326.1	20
**Phenylalanine***	165.19	336.1	30
**Arginine***	147.20	345.1	30
**Tyrosine***	181.19	352.1	30
**Tryptophan***	204.23	375.1	30

*Amino acids were analyzed as AccQTag (6-aminoquinolyl-N-hydroxysuccinimidyl carbamate) derivatives.

Table 5. Patients' characteristics.

Patient No.	Sex	Age	Azathioprine dose per day
1	Female	39	125 mg
2	Female	33	50 mg
3	Female	72	50 mg
4	Female	30	100 mg
5	Male	30	100 mg
6	Male	41	50 mg
7	Male	35	100 mg
8	Female	42	50 mg
9	Female	23	100 mg
10	Male	31	100 mg
11	Female	68	50 mg
12	Female	35	100 mg
13	Female	46	100 mg

Table 6. Determined creatinine concentrations in the human urine samples.

Patient No.	Creatinine concentration (mM)	Volunteer No.	Creatinine concentration (mM)
1	20.80	1	9.41
2	0.262	2	7.19
3	< 0.250	3	42.60
4	13.60	4	24.70
5	5.45	5	6.06
6	2.40	6	2.84
7	23.40	7	20.50
8	3.80	8	6.99
9	22.90	9	32.20
10	13.50	10	1.96
11	1.13		
12	4.32		
13	4.87		

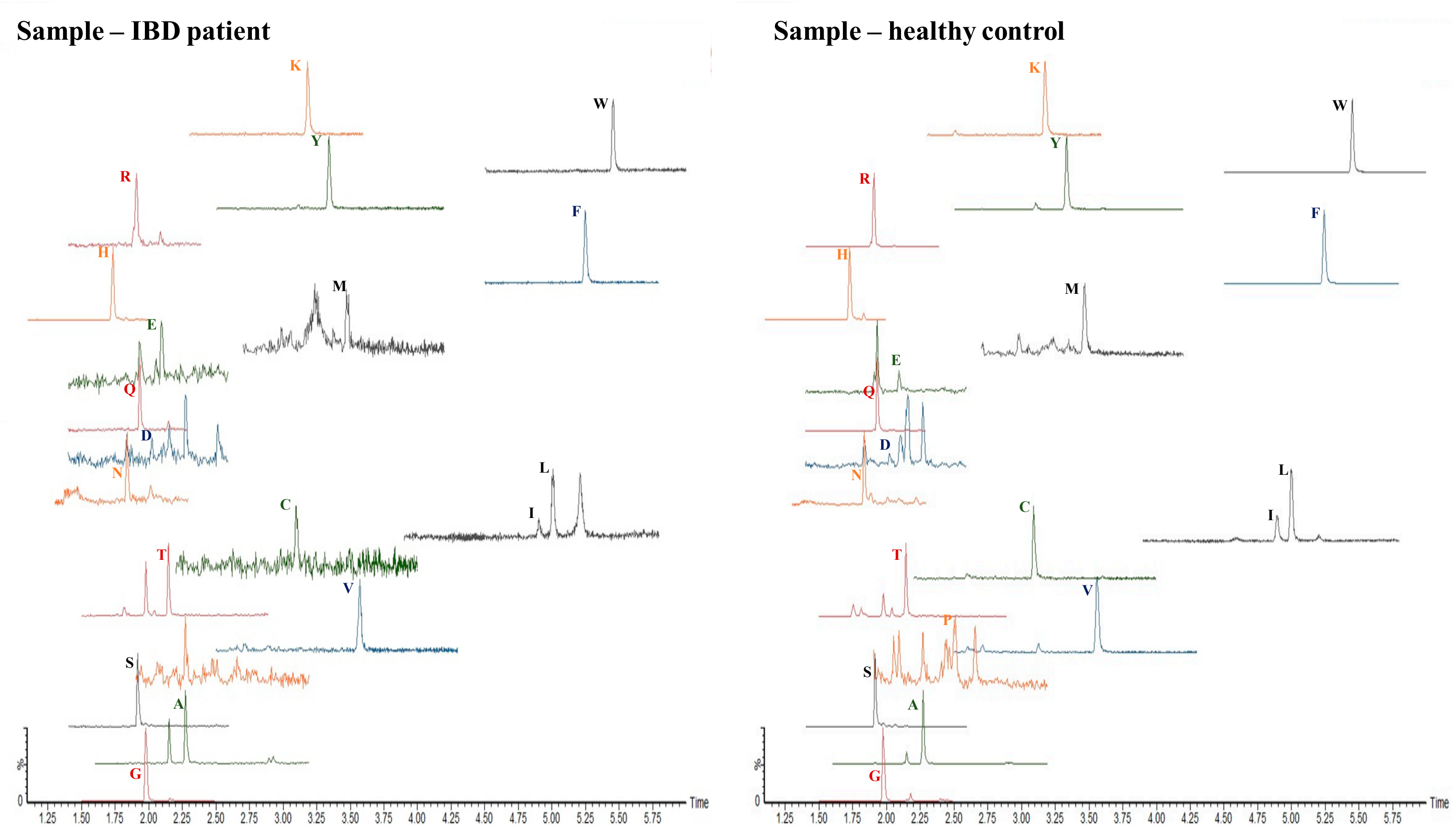

Figure 1. Selected ion chromatograms of derivatized amino acids provided by the UHPLC-MS analysis of clinical human urine samples obtained from a patient suffering from inflammatory bowel disease (left panel) and from a healthy control (right panel). For the sample preparation and other working conditions, see the section 3.
